# Prevalence of Smoking and Related Risk Factors among Physical Education and Sports School Students at Istanbul University

**DOI:** 10.3390/ijerph9030674

**Published:** 2012-02-23

**Authors:** Tümer Ulus, Eray Yurtseven, Bilge Donuk

**Affiliations:** 1 İstanbul University, Cerrahpasa Medical Faculty, Public Health Department, 34320 Kocamustafa Pasa, Istanbul, Turkey; Email: tumerulus@gmail.com; 2 Istanbul University, Physical Education and Sports School, 34310 Avcılar, Istanbul, Turkey; Email: bilgedonuk@gmail.com

**Keywords:** smoking prevalence, related factors, sports, university students

## Abstract

The purpose of this study was to evaluate smoking prevalence and factors associated with smoking among students at the Physical Education and Sports School of Istanbul University. A cross-sectional study was performed on total of 373 students who have been continuing their education at the school from February to March 2011. A total of 166 responders were male (44.5%) and 207 responders were female (55.5%) out of 373 participants. Of the 373 students, 94 (25.2%) were current smokers and the average age for beginning smoking was 18.03 ± 2.6 (min: 12–max: 30). In this study, we found that the smoking prevalence associated with some variables such as age place of residence, mother’s education, father’s education, cigarette or tobacco use in the living place, knowledge status of students about their teacher’s smoking habits and alcohol consumption (*p* ≤ 0.05). These findings suggest that the students, who will train the sportspeople of the future, and should be considered a role model of healthy behavior in society. Consequently, we believe that sports school students should take an active role in providing health education programs to increase their awareness about the detrimental effects of smoking and to extensively quit smoking in public.

## 1. Introduction

Tobacco use is one of the most important worldwide public health problems, in particular for developing countries. It has been reported that among all risk factors smoking is the fourth leading cause of disease [1]. The use of tobacco and its products have gradually increased in the World because of its legal use and easy access. The expansion has been faster, especially among young people, in developing countries [2,3]. Cigarette addiction is one of the most important preventable causes of mortality and morbidity [4,5]. It is known that there are some 1.3 billion smokers worldwide and it is estimated that this number will reach 1.7 billion by 2025, if no intervention is performed. Half the individuals who begin to smoke and keep it up on a regular basis, die as a result of smoking [6]. According to the data released by the World Health Organization (WHO) it has been estimated that 10 million people (70% in developing countries) will die from a disease due to smoking in 2020 if the necessary precautions are not taken. In addition, a person loses their life because of tobacco-related health problems every second [6,7]. It has been reported that there has an increase of smoking, especially among women, adolescents and young adults [8,9]. In Turkey, it appears that although the prevalence of smoking among men has decreased slightly in recent years, it has increased among young adults, especially females [10,11,12,13,14,15,16,17,18,19]. About half of the adult population in Turkey (17 million people) smokes cigarettes, according to the survey conducted by the Thoracic Association. Individuals who smoke in this population have spent $40 million a day and $15 billion per year for cigarettes. Approximately 100,000 people die of smoking-related diseases each year in Turkey. The annual economic cost of smoking-related diseases in Turkey is $2.72 billion [6,7,8,9,10,11,12,13,14,15,16,17,18,19,20,21,22,23,24,25,26,27,28]. A brief survey called the Global Adult Tobacco Survey (GATS) conducted by the WHO in 2009 found that the prevalence of smoking was 40% among people 25–44 years of age, and the prevalence of smoking was 31% among people ≥15 years of age in Turkey. It has been also revealed that more than half of adults in Turkey were non-smokers and 95% of these adults were aware of the existence of warnings on cigarette packets in the same study [10]. The detrimental effects of smoking and using tobacco on health have been investigated for over 50 years. Although the earlier studies reported that smoking cigarette caused lung cancer, the findings of recent studies have proved that around the World the most common diseases among humans, such as coronary heart disease, chronic lung disease and other types of cancer result from the adverse effects of smoking [7,10,11,12]. Smoking habits in young people may be triggered by social and psychological factors such as parental smoking, mimicking others, resistance to authority, affectation to groups of friends, smoking by role models, school failure, poor socioeconomic status, family conflicts and lovelessness, loneliness, alcohol and drug consumption and stress. It should not be forgotten that children’s family, school and friends environment training is also required to prevent children from initiating smoking [13,14,15,16,17]. Educated individuals who have studied physical education have important responsibilities for preventing smoking in society. This study was initiated to investigate the prevalence of smoking and attitudes towards smoking among Physical Education and Sports School students at Istanbul University.

## 2. Material and Methods

This descriptive and cross-sectional study was performed between February and March 2011 at the İstanbul University School of Physical Education and Sports. The data used in this study were obtained through a self-administered questionnaire with 40 items to determine participants’ socio-demographic characteristics, their knowledge, attitude and behaviour towards smoking. The survey data were collected from all students in the school (N = 402) and no sampling method was used. A multiple-item questionnaire was applied to students using the “answering-under-supervision” technique during lessons. The permission for the research was obtained from Directorate of Physical Education and Sports School of Istanbul University. Verbal informed consent was obtained from participants during the implementation of the survey. In this study, a total of 373 out of 402 students responded, for a response rate of 92.7%. In this survey, exclusion criteria included subjects having health problems, absent from school on the day of the study, on vacation, and refusal to participate in the survey. Three questions related to the classification of smoking status were included into the questionnaire. These questions were adapted to Turkish from a U.S. version for the classification of smoking status [18]. The questionnaire items for students and their responses were as follows

1. Have you smoked a total of 100 cigarettes (five packs) in your life? (Yes/No)
2. Do you still smoke? (Yes, every day/less than once a day or more than once a week/No)

Participants who answered the first question with “no” were defined as “never smokers” and all of the others were defined as “smokers”. The second question consisted of three different responses in the present study, although it was consisted a two response item (Yes or No) in the U.S. version. Smokers who answered the second question with “less than once a day or more than once a week” were defined as “eversmokers”. Smokers who answered the second question with “no” were defined as “ex-smokers” [19]. The data obtained in this descriptive and cross-sectional study were given as mean ± standard deviation and percentages. Statistical analysis were performed using the SPSS version 15.0 software package [20]. The chi-square test was used to analyse for significant associations between sex, age, type of high school graduated, place of residence, mother’s education, father’s education, cigarette or tobacco use in the living place, knowledge status of students about their teacher’s smoking habits and alcohol consumption. All statistical analysis was done at 95% confidence level (*p* ≤ 0.05 was considered significant).

## 3. Results

From the 373 respondents who participated in the study, 166 (44.5%) were male and 207 (55.5%) were female. The mean age of respondents was 22.27 years (standard deviation, 2.26 years; range, 18–35 years). The prevalence of smoking among 373 respondents was 25.2% (94 persons). The mean age of smoking initiation was found to be 18.03 ± 2.6 years. The participants had mostly graduated from public (89.3%) school, while 16.1% had graduated from private high schools. Socio-demographic characteristics and factors associated with smoking habits of the participants are shown in Table 1. Univariate (chi-square test) analysis revealed that the prevalence of smoking significantly associated with the factors such as age, place of residence, mother’s education, father’s education level, cigarette or tobacco use in the living place, knowledge status of students about their teacher’s smoking habits and alcohol consumption (*p* ≤ 0.05). It was found that the prevalence of smoking was significantly higher among students who consumed alcohol and those who knew that their teacher smoked. Prevalence of smoking did not significantly differ by sex and classroom grades (*p* > 0.05). Prevalence of smoking decreased as mother’s education increased.

**Table 1:** Socio-demographic characteristics and smoking status of students.

Variable	Smoking Status						
	Smoker		Non-Smoker		Total		*p* Value
	N	%	N	%	N	%	
**Gender**							
Male	44	46.8	122	43.7	166	44.5	*p*: 0.864
Female	50	53.2	157	56.3	207	55.5	
**Grade**							
1th	33	35.1	93	33.3	126	33.8	*p*: 0.289
2th	17	18.1	71	25.4	87	23.3	
3th	25	26.6	53	19	78	20.9	
4th	19	20.2	62	22.2	81	22.1	
**Age (years)**							
18–20	13	13.8	68	24.3	81	21.7	*p* ≤ 0.05
21–23	53	56.4	149	53.4	202	54.2	
24–26	22	23.4	55	19.7	77	20.6	
>27	6	6.4	7	2.6	13	3.5	
**Type of graduated high school**							
Public	83	88.3	230	82.4	313	83.9	*p* ≤ 0.05
Private	11	11.7	49	17.6	60	16.1	
**Place of residence**							
Living with family	82	87.2	236	84.5	318	85.3	*p* ≤ 0.05
Living with friend	4	4.3	18	6.4	22	5.9	
Hostel	4	4.3	19	6.5	23	6.2	
Living alone	4	4.3	6	2.6	10	2.7	
**Mother’s education**							
Illiterate	5	5.3	27	9.6	32	8.6	*p* ≤ 0.05
Primary school degree	31	33	95	34	126	33.8	
High school graduate	19	20.2	65	23.2	84	22.5	
Undergraduate	34	36.2	76	27.2	110	29.5	
Postgraduate	5	5.3	16	6	21	5.6	
**Father’s education**							
Primary school degree	25	26.6	95	34.0	120	32.2	*p* ≤ 0.05
High school graduate	29	30.8	46	16.4	75	20.1	
Undergraduate	23	24.5	108	38.7	131	35.1	
Postgraduate	17	18.1	30	10.9	47	12.6	
**Cigarette or tobacco use in the living place**							
Not smoking	4	4.3	49	17.5	53	14.2	*p* ≤ 0.05
Smoking in a special area	62	66	193	69.1	255	68.4	
Smoking in every place	28	29.8	37	13.4	65	17.4	
**Knowledge status of students on their teachers’ smoking habits**							
Students who know	92	97.9	232	83.1	324	86.9	*p* ≤ 0.05
Students who do not know	2	2.1	47	16.9	49	13.1	
**Alcohol consumption**							
Yes	60	63.8	90	32.2	150	40.2	*p* ≤ 0.05
No	34	36.2	189	67.8+	223	59.8	

The opinions of students on smoking were shown in Table 2. A total of 327 (87.7%) respondents reported that the law for restriction of smoking indoor spaces has been required. 45.3% of the participants believed that the scenes showing smoking should be forbidden, while 131 (35.1%) of the students thought that these restrictions shouldn’t be necessary in the film and TV programs. 

**Table 2:** Attitudes of the students on smoking.

Attitude	Number (n)	Percentage (%)
**On the law of smoking forbidden in all indoors**		
Necessary	327	87.7
Not necessary	19	5.1
Not important	27	7.2
**Application of laws associated with smoking or tobacco use in the scenes of films and movies**		
Necessary	169	45.3
Not necessary	131	35.1
Not important	73	19.6
**Application of health risks of smoking warnings on cigarette packets**		
Sufficient	96	25.7
Insufficient	191	51.2
Not important	86	23.1
**On the prices of cigarette or tobacco**		
More expensive	178	47.7
Suitable	29	7.8
More cheaper	40	10.7
No effect	126	33.8
**Opinions on selling cigarette to the children under the age of 18**		
Not sold	343	92
Parent’s commission	12	3.2
Free	18	4.8
**Having previous training about health risks of smoking**		
Yes	111	29.8
No	262	70.2
*What do you think about, whose mission convince to public for smoking cessation?		
Doctor	177	22.3
Teacher	138	17.4
Sportspeople	119	15
Parents	165	20.8
Friends	128	16.2
Anybody has a mission	65	8.3
* More than one choice were marked by respondents (n = 373) who answered this question.		

51.2% of the students reported that health warnings on cigarette packages were not sufficient. The respondents believed that the massage of which diseases can be caused by smoking should be indicated on cigarette packages. Another interesting result in the present study was that 126 (33.8%) respondents believed that cigarette prices were not considered by smokers. 92% of students thought cigarettes certainly shouldn’t be sold to children under the age of 18. The number of the participants who reported that doctors should convince the smokers to quit smoking was 177 (22.3%), while 119 (15%) respondents indicated that the important task should be conducted by sportspeople, as shown in Table 2. In the present study, the smoking habits and complaints reported by students who smoke (n = 94) are given in Table 3.

**Table 3:** Behaviours and complaints associated with smoking among smokers (n = 94).

Behaviour	Number (n)	Percentage (%)
* Causes of smoking initiation		
Friends’ influence	38	34.8
Affectation	17	15.5
Curiosity	24	22
Family conflicts	5	4.5
School conflicts	15	13.7
Loneliness	10	9.5
**Complaints associated with smoking**		
Dyspnea	16	11.6
Cough	22	16.1
Pharyngitis-sinusitis	6	4.3
Decreasing physical activity	16	11.6
Mouth wounds	4	2.9
Headache	10	7.2
Bad smell of cigarette	51	37.2
No complaint	15	9.1
**Do you want to quit smoking?**		
Yes	66	70.2
No	28	29.8
* More than one choice was marked by respondents (n = 94) answered this question.		

## 4. Discussion

Tobacco and its products were the most prevalent substance used, as in the previous studies performed both in Turkey and all over the World. In Turkey, prevalence of smoking has been found 33.4% among the individuals aged ≥18 years according to the report released by the WHO in 2009 [21]. There have been various studies using different methodologies to determine the prevalence of smoking. In a study conducted by Dabak *et al*. it was reported that the prevalence of smoking was 29.2% in Samsun [22]. Another survey performed by Metintas *et al*. found that the prevalence of smoking in Eskisehir was 42.5% [23]. Onadli *et al*. have reported that the prevalence of smoking was 47.0% among students in School of Tobacco Expertise [24]. The present study found that the prevalence of smoking was 25.2% among the students in School of Sports. However, as compared with the prevalence of smoking reported in the other studies and the WHO report, the value obtained in our study was certainly low among students. The lower prevalence may be a result of the fact that our participants plan to be Physical Education trainers in the future. In fact, it is obviously clear that smoking status and sports are two opposite habits that shouldn’t coexist. Smoking decreases sport performance by affecting the respiratory systems negatively as well as it has destructive effects on most systems and vital organs. In the short term future smoking has a worse image in this group and causes bad breath, dry skin and lower sport performance. Ilhan *et al*. revealed that the prevalence of smoking among males was significantly higher than among females [3]. In the other surveys of medical students, Atilla *et al*. have reached similar findings [2]. 

Although in the present study we determined that the prevalence of smoking among female was higher than among males, the difference was not statistically significant. An association between smoking habits and the factors of environment and friends have been also well documented previously [25,26]. It is clear that smoking initiation has been facilitated among students having friends who smoke and being under pressure of a group of friends. Valente *et al*. reported that 93.1% of smokers initiated smoking with friends [27]. In another study conducted by Ilhan *et al*. it was revealed that 36.7% of the smokers have begun smoking by reason of “friends’ effect”, 19.0% “affectation”, and 18.1% “curiosity” [3]. The present study found that the factors associated with the reasons of smoking initiation were friends’ effect, curiosity, affectation, school conflicts and loneliness (34.8%, 22.0%, 15.5%, 13.7%, 9.5%, respectively). In general, the age of smoking initiation among students has been equalized to the period after graduating from high school. Most of the students have encountered some psychosocial events such as “having new environment”, “having new friends”, “winning recognition in school environment” promoting smoking initiation during the sensitive period. In our study, we found that the variables significantly associated with the prevalence of smoking among students were age, place of residence, mother’s and father’s education, cigarette or tobacco use in the living place, knowledge status of students about their teacher’s smoking habits and alcohol consumption. Previous studies have found that there was a significant association between smoking initiation of students and family’s socioeconomic status and education level [8,9,13,28] but Assanelli *et al*. showed that no difference was found with respect to age with smoking [29]. This finding was different from ours. In our study with increasing age and school grade, the smoking habit was reduced. We have estimated that it was an effect of sports activity, of competition and caused by the fact that lung function is progressively decreasing with the number of smoked cigarettes, then the subject’s performance is impaired.

Princci *et al*. revealed that the prevalence of smoking was higher in students having high parents’ education level than those of low [30]. Atilla *et al*. have shown that there is an association between smoking status and the type of high school graduated [2]. The findings of the present study showed similarities with the literature. We found that the factors associated with smoking initiation at early ages were social environment, use of substances, parent’s smoking, identification with famous individuals such as artists or athletes. In this respect, when the data obtained were evaluated, it is recommended that the individuals who are role models in society, especially for students, shouldn’t smoke cigarettes. In our study, no relation was found in terms of gender and grade of classroom. The majority of students thought that smoking should not be indoors and in addition to the warning of “harmful” found on cigarette packages, it must be specified what other diseases are caused by smoking. Students also believed that cigarettes should be expensive and cigarette sales to individuals under the age of 18 be strictly forbidden to decrease smoking. Less than half of the students considered that the implementation of bans for the scenes with smoking in the movies and TV series must be required. The results of the current study were consistent with the study of Atilla *et al*. Intensive anti-smoking efforts in developed countries have decreased gradually the frequency of smoking in these countries [2,6,10]. It is considered that cigarettes are cheaper in developing countries such as Turkey, so individuals in every age group can easily obtain cigarettes and insufficient smoking cessation campaigns are the most significant reasons for this situation. In our study, the findings reached supported this assessment. Nighty two percent of participants reported that cigarettes should certainly not be sold to people under the age of 18. One of the important findings in the literature is that the vast majority of the students knew the harmful effects of smoking [31]. These previous results were similar to the findings in the present study. In our study, another prominent result was that 70.2% of the students had not received any training before on health hazards of smoking. Therefore, we consider that the topic of health hazards of smoking should be included in the curriculum from the beginning of primary education. Another remarkable result in the present study was that only 22.3% of students reported that individuals should be convinced by the doctors as a professional group to quit smoking. Students thought that this social mission should be implemented by parents, teachers, friends and sportspeople, respectively. This finding may be interpreted that the students have been avoiding assuming responsibility for this important task completely.

## 5. Study Limitations

The findings of the present study had certain limitations. The first limitation of our study was that smoking habits and related to risk factors of the students were determined by using self-administrated questionnaire without physiological or biochemical measurements. Thus, the prevalence of smoking found in the study, may have been influenced by recall bias. Comparing with the literature, the low rate of smoking prevalence could be result from the responders may have underestimated their own smoking behaviour because of the idea that physical education and sports students should not smoke. The second limitation was that the type of study design. It is known that the cross-sectional design does not allow for evaluating of causal relationships between smoking status (dependent variable) and factors (independent variable) associated with smoking. Because of these limitations, we couldn’t say that factors found in our study to be associated precisely with smoking. 

## 6. Conclusions

Students of physical education and sports school know that smoking has detrimental effects on health status. However, we found that more than a quarter of students were smokers in the present study. Considering that the vast majority of students have not received any training on the hazards of smoking, we suggest that health education programs related to smoking should be introduced into the academic curriculum both for increasing their knowledge level on this issue and decreasing those of smoking habits. Special courses might be organized for sport students about the hazards of smoking. Especially we recommend that a lesson should be taught related to smoking and health issues. In addition, these programs should be supported together with post-graduate training to foster antismoking habits. According to our study results, friends’ influence was the foremost factor for smoking initiation. Therefore, smoking cessation campaigns should be planned for groups of friends not the individuals. Students should be given more active role in smoking cessation campaigns and their awareness should be increased. Thus, the students who will be educating the sportspeople of the future can be a role model for the society in terms of health promotion. 
